# Interplay of environmental signals and progenitor diversity on fate specification of cortical GABAergic neurons

**DOI:** 10.3389/fncel.2015.00149

**Published:** 2015-04-28

**Authors:** Juliana A. Brandão, Rodrigo N. Romcy-Pereira

**Affiliations:** Brain Institute, Federal University of Rio Grande do NorteNatal, Brazil

**Keywords:** interneuron, cortical development, non-autonomous specification, inhibitory circuit, cell identity

## Abstract

Cortical GABAergic interneurons constitute an extremely diverse population of cells organized in a well-defined topology of precisely interconnected cells. They play a crucial role regulating inhibitory-excitatory balance in brain circuits, gating sensory perception, and regulating spike timing to brain oscillations during distinct behaviors. Dysfunctions in the establishment of proper inhibitory circuits have been associated to several brain disorders such as autism, epilepsy, and schizophrenia. In the rodent adult cortex, inhibitory neurons are generated during the second gestational week from distinct progenitor lineages located in restricted domains of the ventral telencephalon. However, only recently, studies have revealed some of the mechanisms generating the heterogeneity of neuronal subtypes and their modes of integration in brain networks. Here we will discuss some the events involved in the production of cortical GABAergic neuron diversity with focus on the interaction between intrinsically driven genetic programs and environmental signals during development.

## Introduction

In mammals, the ability to produce their behavioral repertoire relies on a distributed network of neurons that coordinate the action of cortical, subcortical, and spinal cord circuits. From sensorimotor integration to executive functions, all depend on a precise spatio-temporal control of excitation and inhibition in local and long-range networks (Buzsáki, 2010). During brain development, cortical circuits are organized in a well-defined topology of interconnected excitatory and inhibitory neurons whose activities generate coherent behavioral outputs. Inhibitory neurons, in particular, play a crucial role fine-tuning neuronal firing to network oscillations and coordinating the emergence of task-relevant cell assemblies. In addition, GABAergic cells are important to promote the appropriate balance between excitation and inhibition required to prevent over-excitability and excitotoxicity (Somogyi and Klausberger, 2005). Disruption of this inhibitory control has been associated with several neurological disorders including autism, epilepsy, Down syndrome, Fragile X syndrome, X-linked lissencephaly with abnormal genitalia (XLAG), Rett syndrome and schizophrenia (Powell et al., 2003; Levitt et al., 2004; Guidotti et al., 2005; Ramamoorthi and Lin, 2011).

In the adult brain, cortical GABAergic interneurons constitute an extremely diverse population of cells comprising approximately 15–20% of all neurons depending on the cortical region. During the last 20 years, many studies have characterized and classified interneurons based on morphological and functional properties (DeFelipe, 1993; De Felipe et al., 1997; Kawaguchi and Kubota, 1997; Markram et al., 2004; Ascoli et al., 2008; Klausberger and Somogyi, 2008; Le Magueresse and Monyer, 2013). Although some of these properties are defining features, it is interesting to notice that at least some of them do not remain unchanged during lifetime. Studies have shown that some interneurons are able to change electrophysiological properties or the expression of endogenous molecular markers in response to environmental signals, resulting in functional plasticity in response to particular physiological demands (Steriade et al., 1998; Ascoli et al., 2008). Despite this complexity, accumulating evidence has suggested that none of these features alone can unambiguously define a homogeneous population of inhibitory neurons. In addition to the recent efforts to combine these elements and establish a common criterion to classify interneuron subgroups (Ascoli et al., 2008; DeFelipe et al., 2013), significant progress has been made characterizing the spatial distribution of subtype-specific GABAergic progenitors in the rodent embryo and its molecular determinants. Fate mapping experiments have revealed a group of segregated territories in the embryonic ventral telencephalon (subpallium) capable of generating all major populations of cortical GABAergic interneurons. In these proliferative niches, the patterned expression of transcription factors organize the early commitment of progenitors to specific neuronal fates (Parnavelas et al., 1991; Anderson et al., 1997; Sussel et al., 1999; Nery et al., 2002; Schuurmans and Guillemot, 2002; Xu et al., 2004; Wonders and Anderson, 2006; Flames et al., 2007; Guillemot, 2007; Miyoshi et al., 2010; Gelman et al., 2012). The combination of genetic fate mapping and cell transplantation studies have further revealed the contribution of environmental cues to the post-mitotic stages of interneuron specification thought to occur during migration, lamination, and establishment of synaptic contacts (Magrassi et al., 1998; Wichterle et al., 2001; Nery et al., 2002; Valcanis and Tan, 2003; Butt et al., 2005). In this review, therefore, we will discuss the mechanisms responsible for generating the diversity of GABAergic neurons during development and the interactions between environmental signals (extrinsic cues) and genetic programs (intrinsic factors) required to determine early (molecular) and late (molecular plus functional) cell identity, which will combine a proper laminar integration and functional maturation into specific inhibitory circuits.

## Diversity of GABAergic Cortical Interneurons

Cortical GABAergic neurons are aspiny or sparsely spiny non-pyramidal cells that express the GABA-synthesizing enzyme, glutamic acid decarboxylase (GAD). They are found across all cortical layers and establish unique connections with excitatory and inhibitory cells in their vicinity. Some of these cells extend long horizontal or vertical axon collaterals within the cortex and have their cell bodies mostly dispersed into layers II–VI. In the adult brain, inhibitory synapses are precisely organized and contact specific sub-cellular compartments (soma, dendritic shafts, dendritic spines, axon initial segment, and pre-synaptic bouton) of particular neuronal types. This synaptic architecture, once formed, establishes the wiring pattern of local inhibitory circuits (Markram et al., 2004; Somogyi and Klausberger, 2005). Understanding cortical circuit function thus, requires a detailed appreciation of the diversity of GABAergic cells wired in the structure and their pattern of neuronal activity.

Some features can be used to define sub-types of GABAergic neurons: the somato-dendritic morphology, axonal arborization arrangement, post-synaptic sub-cellular target, biochemical identity, intrinsic electrophysiological properties, and modes of synaptic and structural plasticity (Gupta et al., 2000; Markram et al., 2004). As for their perisomatic and axonal arborization, cortical GABAergic interneurons are frequently identified as multipolar, bipolar or bitufted, chandelier, basket, or neurogliaform cells with axonal ramifications targeting distinct inhibitory domains on post-synaptic neurons. Basket cells for example show a perisomatic pattern of innervation establishing axo-somatic and axo-dendritic symmetric contacts. In contrast, chandelier cells (or axo-axonic interneurons) contact exclusively the axon initial segment and multipolar Martinotti cells innervate distal dendrites of pyramidal neurons. Such variety of synaptic sites, aiming distinct sub-cellular compartments allows a precise inhibitory control over inputs from different cortical layers.

The molecular composition of GABAergic neurons is also diverse with the expression of calcium-binding proteins parvalbumin (PV), calretinin (CR) and calbindin (CB), and neuropeptides, such as somatostatin (SST), neuropeptide-Y (NPY), cholecystokinin (CCK) and vasoactive intestinal peptide (VIP; Houser et al., 1983; DeFelipe, 1993; Kawaguchi and Kubota, 1997; Gonchar, 2008). Other biochemical markers such as nitric oxide synthase (nNOS), reelin, serotonin receptor 3A (5HTR-3A) are also found in interneuron subpopulations such as ivy cells (neurogliaform), Cajal–Retzius neurons (transient embryonic layer I cells) and superficial layer neurons co-expressing VIP, respectively (Vucurovic et al., 2010; Jaglin et al., 2012). However recent findings have shown that three molecularly distinct non-overlapping populations of neurons can be sorted out according to the expression of PV, SST, and the ionotropic 5HTR-3A (Lee et al., 2010; Rudy et al., 2010). Interestingly, these neurons are born from spatially distinct domains of the embryo.

Interneurons also display a great diversity of intrinsic electrophysiological properties characterized by distinct firing modes in response to input stimuli or step-current injections. They can be classified according to their broad electrophysiological properties as non-accommodating (∼50%), accommodating, (37%) and stuttering cells (∼13%) that can be further sub-grouped into classical spiking (regular fast-spiking and non-fast-spiking), bursting, and delayed onset response cells. Overall, it can be identified at least 14 individual functional sub-types of inhibitory cells (Gupta et al., 2000; Ascoli et al., 2008). Nevertheless, it is important to be cautious when considering this as static properties of a neuronal type. There still little information on inhibitory firing mode plasticity in animals *in vivo*.

Parvalbumin– and somatostatin-expressing interneurons are the two most abundant classes of cortical interneurons with non-overlapping molecular identities and relatively large cell bodies (>20μm; DeFelipe, 1993, 1997; Kawaguchi and Kondo, 2002). GABAergic interneurons expressing PV make up ∼40% of all cortical interneurons of which basket and chandelier cells are typical members. These cells do not express SST, VIP, or CCK, but have some overlap labeling with CR and CB. They emit axonal collaterals to the soma, perisomatic dendrites and axons of post-synaptic targets. Functionally, these neurons show low-input resistance and fast-spiking dynamics of non-accommodating short-duration action potentials that impart a strong inhibitory control over their post-synaptic targets (Kawaguchi and Kubota, 1997; Gupta et al., 2000; Massi et al., 2012). PV-expressing inhibitory neurons represent an important population of cells involved in the modulation of critical period of auditory plasticity and implicated in psychiatric disorders, such as schizophrenia (Beasley and Reynolds, 1997; de Villers-Sidani et al., 2008). SST-expressing neurons, on the other hand, comprise ∼30% of the cortical inhibitory cells with soma preferentially distributed across deep cortical layers V–VI and axons branching into layer I where they exert inhibitory control over distal dendritic processes of pyramidal neurons. Martinotti cells, the most studied sub-population of SST-positive neurons so far, have multipolar somato-dendritic morphology and either regular adapting or intrinsically bursting activity. However, SST is also expressed in three other cell types: the murine X94 transgenic cell line located in layers IV–V with projections to layer IV and either short-duration spikes or stuttering firing pattern (Ma et al., 2006) and, the cells named group 2–3 lines of short asymmetric axons that project to layers II/III. Yet they differ in their electrophysiological properties as one displays strongly adapting regular or stuttering firing modes, whereas the other have regular firing with narrower action potentials (McGarry et al., 2010). SST-positive neurons can also co-express CR (21%), NPY (7%), nNOS, reelin, or CB in various proportions (Pesold et al., 1999; Xu et al., 2010b; Jaglin et al., 2012). The third class of cortical inhibitory neurons with non-overlapping profiles with PV- and SST-positive cells expresses the ionotropic serotonin receptor 5HTR-3A. These cells comprise ∼20–30% of GABAergic neurons in the somatosensory cortex and represent the largest group of superficial neocortical interneurons (Morales and Bloom, 1997; Xu et al., 2010b). Despite the heterogeneity of co-labeling with CCK, NPY, and nNOS, two main subtypes of 5HTR-3A-expressing neurons can be distinguished: cells expressing VIP (∼40% of all 5HTR-3A cells; located in layers II/III) and VIP-negative cells (∼60%), most of them expressing reelin (∼50%; located in layer I). Lee et al. (2010) showed that more than 50% of VIP cells are of bitufted morphology and respond as irregular spiking neurons, while Miyoshi et al. (2010) described four distinct electrophysiological firing modes among VIP cells: burst non-adapting, delayed non-fast spiking, irregular spiking, and fast adapting cells. In contrast, most of VIP-negative GABAergic neurons are neurogliaform cells (reelin-positive) and fire accommodating delayed onset spikes in response to current steps (Lee et al., 2010).

Despite the great heterogeneity, GABAergic interneurons seem to select a group of neurons of the same class and establish synapses with homogenous temporal dynamics. In addition, a study using electrophysiological recordings obtained from pairs of different post-synaptic neurons following stimulation of the same pre-synaptic cell, showed that the same GABAergic axon can establish different types of synapses on distinct classes of target neurons (Gupta et al., 2000). Altogether, it is remarkable but still elusive, how such diversity is generated from a limited number of ventral telencephalic progenitors in the embryo.

### Diversity of Cortical Interneuron Progenitors

**FIGURE 1:**
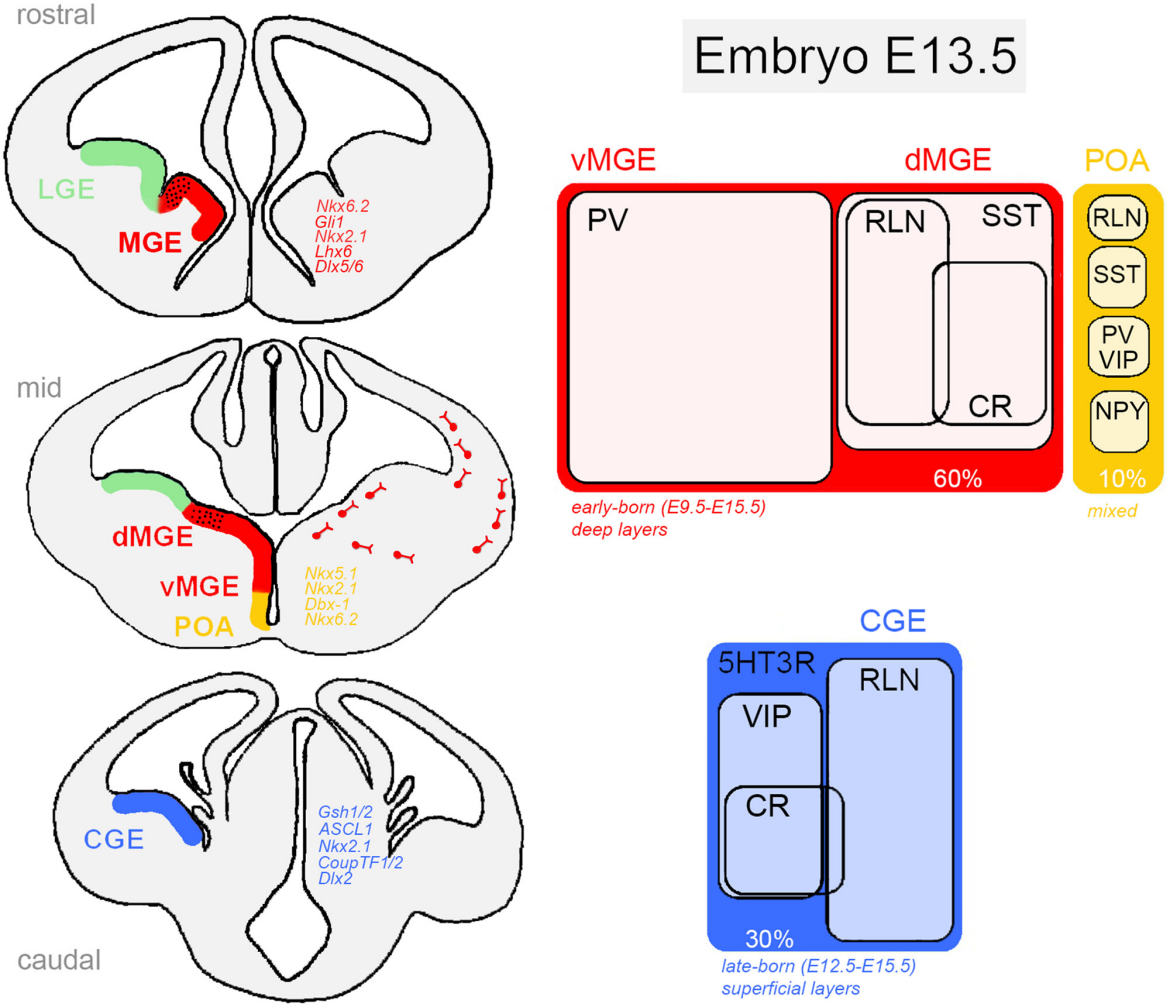
**Ventral telencephalic germinative zones of cortical GABAergic neurons in the rodent embryonic brain**. The medial ganglionic eminence (dorsal, dMGE; ventral, vMGE), caudal ganglionic eminence (CGE), and the preoptic area (POA) are responsible for generating virtually all cortical interneurons. In each region, neural progenitor classes are defined by a combinatorial action of transcription factors **(Left)** that restrict cells to neurochemically defined fates **(Right)**. Distinct classes of inhibitory cells display characteristic morphology, laminar distribution and electrophysiological properties. *5HT3R, serotonin receptor type 3; CR, calretinin; NPY, neuropeptide Y; PV, parvalbumin; RLN, reelin; SST, somatostatin; VIP, vasointestinal active peptide. Genes: ASCL1, achaete-scute family bHLH transcription factor 1 (Mash1, mammalian homologue); Dbx-1, developing brain homeobox 1; CoupTF1/2, chicken ovalbumin upstream promoter transcription factor; Dlx (Dlx1/2/5/6), distal-less homeobox; Gli1, Gli family zinc finger 1; Gsh1/2, GS homeobox 2; Lhx6, LIM homeobox 6; Nkx (Nkx2.1, Nkx5.1, Nkx6.2), NK homeobox family.*

During embryonic development, excitatory neurons are generated in the ventricular zone (VZ) of the dorsal telencephalon (pallium) and gradually invade the cortical plate by radial migration through specialized glial cells (Rakic, 2009). In the cortex, cell identity and laminar distribution are temporally organized by gene expression patterns and time of cell cycle exit (i.e., birthday), respectively (Angevine and Sidman, 1961; Rakic, 1988). In contrast, most of the brain’s GABAergic interneurons derive from progenitors located in four different sub-regions of the subpallium: the medial (MGE), lateral (LGE) and caudal (CGE) ganglionic eminences and, the preoptic area (POA), which are defined by the expression of the homeodomain transcription factor *Dlx1/2* and the absence of *Pax6* gene (**Figure 1**). Neural progenitors localized in the MGE, CGE, and POA give rise to virtually all cortical interneurons, whereas LGE progenitors produce GABAergic cell populations of olfactory bulb, amygdala, and striatum (Van Eden et al., 1989; Nery et al., 2002; Stuhmer et al., 2002; Xu et al., 2004; Flames et al., 2007; Gelman et al., 2011). It is important to mention that excitatory and inhibitory neurons are generated independently, in spatially segregated domains and do not share a common lineage. In the subpallium, regional identity is regulated by a combination of transcription factors with overlapping expression patterns, some of them showing cell fate restriction functions (Anderson et al., 1997; Sussel et al., 1999; Flames and Marin, 2005).

Medial ganglionic eminence progenitors are responsible for generating approximately 60% of all GABAergic neurons in the cortex. Around embryonic day 9 (E9), the action of the signaling molecule *Shh* (*sonic hedgehog*) on MGE cells induces the expression *Nkx2.1* and *Lhx6* transcription factors that orchestrates intracellular cascades required for the specification of SST-expressing and PV-expressing interneurons (Butt et al., 2008; Du et al., 2008). Although *Nkx2.1* and *Lhx6* specifically define the MGE neuroepithelium, *Nkx2.1* is only briefly expressed in MGE progenitors. In a cascade of events, *Lhx6*, a direct target of *Nkx2.1*, is required for the acquisition of molecular identity and laminar positioning (Sussel et al., 1999; Liodis et al., 2007) and *Sox6* and *Satb1* seem to act as downstream effectors of *Lhx6* (Azim et al., 2009; Batista-Brito et al., 2009; Close et al., 2012). Analysis of *Lhx6* mutant mice has shown that even though *Lhx6*^-/-^ progenitors are still able to migrate to the pallium, most derived interneurons lack PV and SST expression and show abnormal laminar integration (Zhao et al., 2008). Interestingly, Xu et al. (2010a) have recently demonstrated that high levels of *Shh* in dorsal MGE compared to ventral MGE is responsible for a dorso-ventral patterning of progenitors in this region, suggesting an important role for soluble factors in early fate determination in the subpallium. As a result, two different territories in the MGE can be identified. The dorsal division (dMGE) is enriched in *Nkx6.2* and *Gli1* genes, and preferentially gives rise to SST-expressing interneurons comprising about 65% of all MGE-derived neurons, including Martinotti cells that co-express CR, NPY-expressing cells and all nNOS-positive neurons. The ventral division (vMGE), in contrast, is enriched in *Dlx5/6* and *Lhx6* genes and generates most of the PV-expressing interneurons of the cortex (∼35%) that includes large basket and chandelier cells (Fogarty et al., 2007; Jaglin et al., 2012).

These two lineages of neurons are born around E12.5-E16.5 (peak E14.5) and migrate tangentially toward the pallium through the subventricular zone (SVZ) and marginal zones (MZ) to be subsequently incorporated into the cortical plate (Butt et al., 2005; Miyoshi et al., 2007). They follow a temporal-positioning code according to their birthdate showing an inside–out neurogenic gradient of lamination. Although the final distribution of MGE-derived neurons encompasses preferentially deep cortical layers (layers IV–VI), some neurons have relatively restricted laminar patterns as seen for chandelier cells that prefer layers II and IV (Taniguchi et al., 2013).

The other major contributing region for the generation of cortical GABAergic neurons is the CGE that can be considered the caudal extension of MGE and LGE in the ventral telencephalon. The CGE is responsible for producing about 30% of all adult cortical interneurons, of which virtually all express the serotonin receptor subtype 3a (5HT3aR; Rudy et al., 2010; Vucurovic et al., 2010). Interneurons from the CGE have bipolar or double-bouquet morphology and display electrophysiological characteristics of irregular firing or fast-adapting cells. Electrophysiological studies have grouped these cells in more than six different subtypes according to their firing patterns and morphology (Lee et al., 2010; Miyoshi et al., 2010). Forty percent of these neurons co-express VIP (some also expressing CR, but negative for SST), whereas about 80% of the remaining VIP-negative cells express the extracellular signaling protein reelin (Nery et al., 2002; Lee et al., 2010; Miyoshi et al., 2010; Jaglin et al., 2012). Overall, CR-positive/SST-negative cells, as well as the great majority of VIP-, CCK-, and reelin-expressing GABAergic interneurons are derived from 5HT3aR-expressing CGE progenitors.

These progenitors can also be identified by an abundant expression of the transcription factors *CoupTF1/2*, *Gsh1/2* (Sousa et al., 2009; Lee et al., 2010). *Gsh2* is enriched in the CGE and controls the expression of *Mash1*, *ASCL1*, *Dlx2* required for CGE patterning. In fact, there is evidence suggesting an internal regionalization of the CGE, in which the dorsal region expresses *Gsh2* and induces the proneural gene *Mash1* and *Delta* (a Notch ligand), leading to repression of progenitor differentiation, whereas the ventral CGE is enriched in *NKx2.1*. The consequences of this molecular regionalization are still not clear.

Caudal ganglionic eminence-derived neurons are late-born cells with peak production around E16.5, migrating tangentially toward the pallium through the SVZ and MZ (Cavanagh and Parnavelas, 1989). They do not follow a temporal-positioning code, but instead get distributed homogeneously across superficial cortical layers (layers I–III), with ∼40% occupancy in layers II and III. An exception to this rule is the outside-in neurogenic gradient observed in CR-positive interneurons (Lee et al., 2010).

In the ventro-medial region of the subpallium, the POA generates the remaining 10% of cortical interneurons, including multiple classes of GABAergic cells. They consist of a small fraction of PV-, SST-, NPY-, and reelin-expressing cells, all derived from POA progenitors with transcriptional programs distinct from those of the MGE and CGE (Gelman et al., 2011). Interestingly, mice lacking *Lhx6* expression (a specific marker of MGE progenitors) are almost completely depleted of PV-positive and SST-positive neurons, except for some scattered cells possibly derived from POA, seen in deep layers of the cortex (Liodis et al., 2007; Zhao et al., 2008). Fate determination in the POA seems to be orchestrated by the action of several genes under the instruction of *Shh* and *Nkx2.1*. Using genetic fate-mapping and transcription factor expression analysis, it has been shown that POA contains at least two progenitor domains defined by the non-overlapping cellular expression of *Dbx-1*, *Nkx6.2* (ventrally), and *Nkx5.1* (dorsally; Flames et al., 2007; Gelman et al., 2011). About 40% of the POA-derived interneurons are generated by *Dbx-1*-expressing progenitors, most of them born on E12.5, distributed across layers V and VI of the cortex and expressing PV, SST, and reelin. On the other hand, *Nkx5.1*-derived interneurons are mostly found in superficial layers of the cortex and constitute a rather homogeneous population of rapidly adapting interneurons, many of which express NPY. However, it is still not clear so far how such diversity of interneuron subtypes arise from the *Dbx-1*-expressing lineage (Gelman et al., 2009).

### Role of Environmental Factors on Cortical Interneuron Differentiation

Cortical interneurons acquire much of their adult identity by the time of birth (i.e., after the last cell division), due to early fate restriction imposed by genetic programs (Xu et al., 2005; Flames et al., 2007; Fogarty et al., 2007; Miyoshi et al., 2007; Butt et al., 2008; Azim et al., 2009; Batista-Brito et al., 2009; Neves et al., 2013; Kessaris et al., 2014; Vogt et al., 2014). However, many of the characteristics used to define interneurons are not evident until late postnatal ages and even adulthood. Therefore, it is reasonable to inquire what are the post-mitotic events necessary to assure proper development of cortical inhibitory circuits. Recent findings have given support to the idea that interactions with environmental signals starting from early post-mitotic stages and all way along their migratory path to final destination are essential for the full expression of mature features (Valcanis and Tan, 2003; Rouaux and Arlotta, 2010; De Marco García et al., 2011; Lodato et al., 2011; Close et al., 2012; Denaxa et al., 2012; Spitzer, 2012). Some of the features that undergo protracted specification include cortical area assignment, laminar positioning, neurochemical identity, dendritic arbor topology, and post-synaptic cell targeting.

**FIGURE 2:**
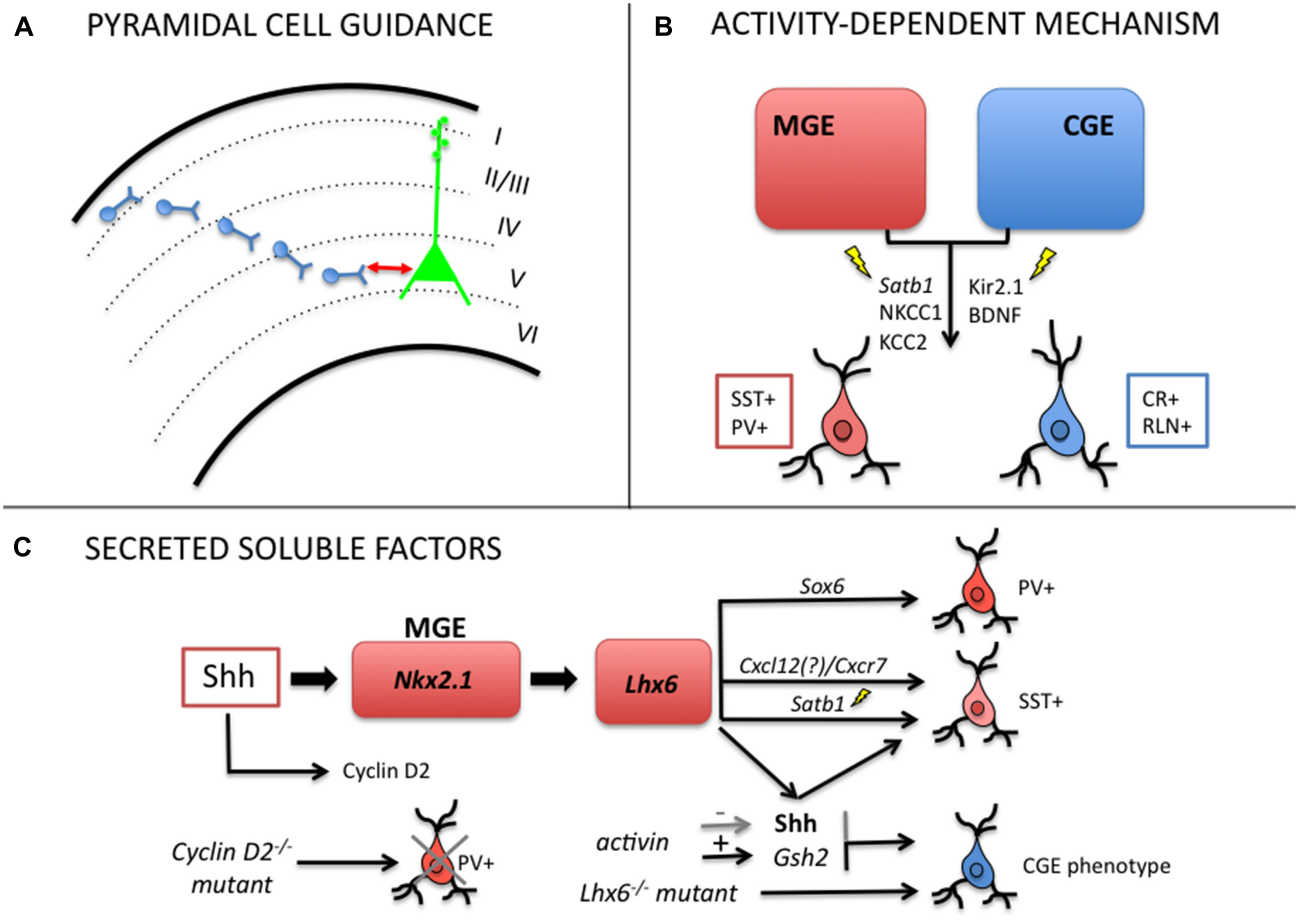
**Mechanisms regulating post-mitotic plasticity and late-fate specification of cortical GABAergic interneurons. (A)** Local pyramidal cells modulate laminar positioning of inhibitory neurons in the cortex (Pla et al., 2006; Lodato et al., 2011). **(B)** Electrical activity (GABA-mediated membrane depolarization, Ca^2+^ transients) can regulate late acquisition of molecular identity in MGE- and CGE-derived interneurons (Berghuis et al., 2004; Borodinsky et al., 2004; De Marco García et al., 2011; Close et al., 2012; Denaxa et al., 2012). **(C)** Soluble secreted factors can alter the balance of interneuron subtypes through shift in neurochemical identity (Xu et al., 2005; Lopez-Bendito et al., 2008; Cambray et al., 2012; Vogt et al., 2014).

Molecular signals in the environment as well as activity-dependent membrane currents seem to be necessary cues to guide the execution and fulfillment of genetic programs set in motion during early stages of interneuron development (**Figure 2**). For instance, evidences show that manipulations of these environmental cues surrounding neural progenitors affect the neurochemical identity of mature interneurons. Resident pyramidal neurons, electrical activity, and local molecular codes are thought to instruct intrinsic programs and promote neuronal migration, layer positioning, cell target selection, and the precise apposition of subcellular synaptic contacts. Here we will discuss some of these studies.

### Pyramidal Cells Controls Interneuron Laminar Identity

Laminar positioning of interneurons in the cortex depends both upon their place of origin and date of birth (Miyoshi and Fishell, 2011), however, evidence points to a more interactive and epigenetic perspective on interneuron laminar specification. Valcanis and Tan (2003) have shown that early- and late-born interneuron progenitors have their laminar fate re-specified by extrinsic signals of the new environment. Using transplantation experiments, the authors show that donor progenitor cells can acquire the host laminar distribution once they undergo a last round of cell division in the host tissue (Valcanis and Tan, 2003).

It was also observed that the interaction of migrating interneurons with resident pyramidal cells control the laminar identity of GABAergic cells. Pla et al. (2006) showed that disrupting the normal layering of pyramidal neurons, in *Dab1-/-* mice deficient in reelin signaling, modifies the laminar allocation of MGE-derived cells. Wild-type interneurons transplanted into the cortex of *Dab1* mutants closely matched the layer distribution of host pyramidal neurons, whereas *Dab1-/-* interneurons transplanted in wild-type cortex were able to generate normal interneuron positioning, as if guided by well-positioned local pyramidal cells (Pla et al., 2006). Collectively, these findings support a reelin-independent mechanism where pyramidal neurons are required for proper interneuron integration. The authors also noticed that more than 20% of early born (E12) and ∼60% of late born (E15) wild-type interneurons change their laminar fate upon transplantation into older (E15) developing wild-type cortex.

In agreement with the previous results, Lodato et al. (2011) have recently shown that SST- and PV-expressing interneurons are specifically reduced in layer 5 of *Fezf2*-depleted mice, in which subcerebral pyramidal projection neurons are absent from the cortex. The lack of glutamatergic projection neurons in layer 5 of these animals creates an abnormal distribution of PV and SST neurons, which suggests that these neurons control the laminar positioning of inhibitory migrating cells in the cortex (Lodato et al., 2011).

### Electrical Activity Instructs Phenotypic, Laminar and Synaptic Identity

Another environmental signal that regulates late fate specification of cortical interneurons is electrical activity. In CGE-derived neurons, normal excitability is required for proper integration into their final destination. Using *in utero* electroporation with the inward rectifying potassium channel Kir2.1 directed to CGE-derived neurons, De Marco García et al. (2011) showed that depolarization was necessary for the full phenotypic development of CR- and reelin-expressing cells. They observed morphological defects in the axonal arbor and dendritic tree of both neuron subtypes or only in reelin-expressing cells under hyperpolarization, respectively. Although tangential migration was unchanged, it was detected a shift in laminar positioning when potassium currents were induced after post-natal day 5 (P5) in CR+ and Re+ CGE-derived neurons. These results indicate that genetic programs initiated at progenitor stage can be modulated by electrical activity during development.

Furthermore, Berghuis et al. (2004) have shown that neuronal depolarization of PV-expressing neurons maintained in culture enhances the BDNF differentiating effects. While BDNF promotes dendritic branching, somatic differentiation, strengthening of synaptic connections, and frequency modulation of action potentials, the addition of KCl (i.e., depolarization) was required for the establishment of reciprocal inhibitory synapses and significantly accelerated the formation of synaptic contacts (Berghuis et al., 2004). Consistent with this idea, GABA and glutamate play an important role in post-mitotic cortical neuronal motility as it modulates intracellular calcium (Ca^2+^) transients through GABAA and NMDA/AMPA receptor-mediated depolarization. In contrast, hyperpolarizing GABA decreases the magnitude of Ca^2+^ transients inhibiting motility of migrating cells. After reaching the cortex, migrating interneurons upregulate the potassium/chloride (K^+^/Cl^-^) exchanger KCC2, which determines the developmental switch from depolarizing to hyperpolarizing action of GABA (Ben-Ari, 2002). KCC2 expression thus can act as a switch to induce a voltage-sensitive, Ca^2+^-mediated reduction of interneuron motility and function as a migration stop signal (Bortone and Polleux, 2009). Indeed, migrating cortical interneurons may sense and integrate the ambient, local extracellular levels of GABA and glutamate as a way to determine when to stop migration. In the ganglionic eminence, progenitors are the main source of GABA and MGE-derived interneurons begin to express KCC2 a few hours after reaching the cortex (Hevner et al., 2004; Inamura et al., 2012).

Ca^2+^ spikes also modulate the neurochemical specification of neural progenitors between GABA/glycine and glutamate/acetylcholine in the dorso-ventral axis of neural tube (Borodinsky et al., 2004). Cells exhibiting high frequency of Ca^2+^ spikes express GABA and glycine, whereas cells with a low spike frequency take the glutamatergic and cholinergic phenotype. In the spinal cord, Ca^2+^-mediated signaling contributes to proliferation, migration, axon pathfinding, dendritic growth, and specification of neurotransmitter subtype (Rosenberg and Spitzer, 2011).

Another particular example concerns the chromatin organizer and transcription factor *Satb1* (special AT-rich binding protein) that is specifically expressed in mature interneurons located in the cortical plate. Two recent studies have demonstrated that *Satb1* is a key molecule acting downstream *Lhx6* to control maturation and late differentiation of cortical interneurons in an activity-dependent manner. Ablation of *Satb1* in mice promotes a dramatic decrease of SST mRNA and protein expression in postnatal cortical interneurons, but no effect on the expression in migrating precursors (Close et al., 2012; Denaxa et al., 2012). Close et al. (2012) showed that embryonic deletion of *Satb1* in MGE-derived interneurons disrupts the migration and synaptic integration of both PV- and SST-expressing neurons into nascent cortical circuits with special effect on the differentiation of SST-expressing cells. Interestingly, *Satb1* is regulated by neuronal activity, which is required for the establishment of mature patterns of inhibition onto cortical pyramidal cells (Close et al., 2012). The second study showed that treatment of dorsal telencephalon cell cultures with KCl induced the expression of *Satb1* and *c-FOS* in cortical GABAergic neurons within 24 h. In addition, *Satb1* was shown to be required for the induction of the neurochemical phenotype typical of mature SST-expressing GABAergic neurons and was regulated by neuronal excitability involving Ca^2+^ influx and GABA receptor activation. Using misexpression experiments in the MGE of E14.5 embryos, the authors observed that *Satb1* could further affect neuronal activity by modulating the expression of *NKCC1* (a Na^+^–K^+^–Cl^-^ co-transporter abundantly expressed in immature GABAergic interneurons) and *KCC2* (Denaxa et al., 2012). *KCC2* expression by its turn could set off the termination of cortical interneuron migration in a voltage-sensitive and calcium-dependent manner.

### Soluble Factors Shape Cell Identity

Another possible mechanism of post-mitotic plasticity is the interaction of migrating interneurons with soluble molecules in the environment. In an early study, Iacovitti et al. (1987) observed that post-mitotic neurons obtained from E13 embryonic cortical cultures, grown for 1 day, expressed a catecholaminergic phenotype different from what was observed *in vivo* in the cortex. The authors then suggested that external factors might have interfered with neurotransmitter specification *in vitro* that did not occur *in vivo* (Iacovitti et al., 1987).

Other studies further revealed that environmental signals could in fact regulate neurochemical identity of GABAergic neurons. In a first report, dissociated cortical interneurons prepared at birth failed to express VIP at P2, whereas interneurons from neonatal slices of same age did express the neuropeptide (Gotz and Bolz, 1994). Following that Gulacsi and Lillien (2003) showed that dorsal telencephalic progenitors co-cultured with ventral progenitors generated more GABAergic neurons than in single culture. This effect was reduced or enhanced by the addition of cyclopamine, an antagonist of *Shh* or exogenous *Shh*, respectively (Gulacsi and Lillien, 2003). New evidence to *Shh* involvement came from Xu et al. (2005) showing that dissociated interneuron cells treated with cyclopamine expressed reduced PV and SST phenotypes. Moreover, using a conditional mutant mice *NestinCre:Shh(flox/flox)*, in which *Shh* signaling is deficient, they found altered MGE patterning and reduced number of NPY, PV, and SST neurons in the somatosensory cortex (Xu et al., 2005). In fact, *Shh* seems to maintain the identity of cortical interneuron progenitors in the ventral telencephalon through a continuous provision of positional information by regulating *Nkx2.1* expression. In this manner, it plays a critical role in determining the relative composition of cortical excitatory and inhibitory neurons.

It is known that post-mitotic GABAergic neurons are guided toward the cortex along two main migratory streams (MZ and SVZ) by the interaction with soluble chemoattractants and chemorepellents dispersed in the pathway. Among them, Cxcl12 (and its receptors, Cxcr4, Cxcr7), a potent chemoattractant to MGE-derived interneurons is required for normal positioning of interneurons in the cortex (Li et al., 2008; Lopez-Bendito et al., 2008). Recently, it was reported that Arx and Cxcr7 (*Lhx6*-target genes) could rescue the cell identity and laminar phenotype of Lhx6^-/-^ MGE cells. In *Lhx6* mutant mice, PV and SST-expressing neurons are specifically reduced and some cells acquire CGE-like fate. *Lhx6/8* are known to be necessary for *Shh* expression in MGE and to determine cell fate of PV- and SST-neurons. Besides, *Lhx6/8* are also required for repressing *Dlx1* in the pallium, preventing GABAergic fate of local progenitors (Flandin et al., 2011; Vogt et al., 2014).

Another example of a soluble molecule with putative fate specification role is activin. It is a member of the TGF-3 family of neurotrophic factors, produced in LGE/CGE and shown to induce telencephalic precursors to adopt LGE/CGE cell fate. Through the specific induction of *Gsh2* expression and inhibition of *Shh* and *Nkx2.1*, it promotes the acquisition of CGE fate and CR phenotype (Cambray et al., 2012). In fact, neurotrophins were already shown to regulate the relative number of GABAergic and cholinergic neurons in the rodent basal forebrain through their action on p75 receptor during development (Lin et al., 2007). Moreover, some studies show that serotonin depletion transiently delays the incorporation of CGE-derived interneurons into the cortical plate and alters the maturation of CR- and CCK-expressing interneurons in the somatosensory cortex. Such depletion also decreases reelin secretion by Caja–Retzius cells leading to dendritic hyper-complexity in pyramidal neurons (Vitalis et al., 2007, 2013).

More recently, Díaz-Alonso et al. (2012), using *in vitro* and *in vivo* experiments, observed that embryonic endocannabinoids (eCB) regulate the intrinsic program for layer fate specification of cortical pyramidal neurons through CB1 receptors. The authors showed that CB1 signaling controls the appropriate balance of neuronal differentiation in deep cortical layers by inhibiting *Satb2* and promoting *Ctip2* expression following extracellular levels of eCB. Cortico-spinal and subcerebral projection neurons were particularly affected. Interestingly, CB1 receptor inactivation decreased the expression of deep-layer markers *Fezf2* and *Ctip2* in pyramidal cells, but did not affect *Ctip2* expression in GABAergic neurons (Díaz-Alonso et al., 2012). However, considering that laminar identity of cortical GABAergic interneurons can be regulated by resident glutamatergic cells, these findings also suggest that eCB might indirectly govern the laminar allocation of interneurons.

Another indirect effect on the final organization of cortical interneuron circuits may be exerted by the neurotrophin 3 (Nt3). Through the repressor *Sip1*, Nt3 acts as a feedback signal between post-mitotic and progenitors neurons. It is able to switch the cell fate of apical progenitors and promote overproduction of superficial layer cortical neurons in the developing mouse. Changing the balance of superficial and deep projection neurons, Nt3 might affect interneurons layer positioning. *Sip1* could also switch progenitors fate from neurogenesis to gliogenesis (Seuntjens et al., 2009; Parthasarathy et al., 2014).

Finally, the number of specific interneuronal subtypes in the cortex and their final fate was shown to be critically determined by the regulation of cell cycle in neuronal progenitors. Actually, it was observed that *cyclin D2* delays cell cycle exit of MGE progenitors and its deletion leads to a prominent reduction in PV-positive cells in the mature rodent cortex along with microcephalic phenotype (Glickstein et al., 2007). Interestingly, *cyclin D2* expression seems to be regulated by Nt3 and Shh (Lukaszewicz et al., 2002; Komada et al., 2013).

## Conclusion

Cell fate specification of cortical interneurons seems to require the interplay of both intrinsic and extrinsic molecular signals. However, the distinct aspects of such delicate control just began to be unveiled. Time of signaling, cell-type targeting, magnitude of phenotypic effects, and the particular molecular mechanisms involved are still unknown. Future experiments should bring some light on these open questions.

## Conflict of Interest Statement

The authors declare that the research was conducted in the absence of any commercial or financial relationships that could be construed as a potential conflict of interest.
